# Surgery

**Published:** 1911-12

**Authors:** Harold F. Mole


### SURGERY.

Anatomically **cervical ribs** are not uncommon. As, however, only a small proportion give rise to symptoms they are clinically rather rare. The symptoms they may give rise to are interesting in themselves, apart from the fact that difficulties and errors in diagnosis are still common. These have been, however, lessened since the advent of the X-rays. It must be remembered that even a skiagram of this condition may be interpreted incorrectly, as mentioned by Howard Williams.[1]

Cervical ribs are usually bilateral, though their symptoms are usually unilateral. The rib may be "floating" with its extremity free somewhere in the neck, or it may have an attachment to the first rib or sternum by bone, cartilage or fibrous tissue. It usually originates from the transverse process of the sixth or seventh cervical vertebra. The symptoms it gives rise to are referable to the nerves amongst which it lies, or to the circulation through the subclavian artery which crosses it. Also sometimes a lump may be complained of in the neck, and the wearing out of the clothes from pressure on this spot. The symptoms are not usually complained of until adult age, Murphy[2] giving twenty-seven years as the average in twenty-nine cases. Spellissy,[3] however, had to operate on a case twenty years of age; Williams on one of eighteen, who had been watched for over a year with symptoms getting steadily worse; and Hessert[4] quotes a case of a girl of sixteen in whom, following a fracture of the clavicle, the cervical rib was found accidentally, it giving rise to no special symptoms. The cervical ribs are at times found associated with other deformities, and Royal Whitman[5] mentions such a case in a boy of ten years, who had also congenital torticollis, spinal curvature and meningocele. The symptoms produced by pressure on the nerve trunks (Murphy[2]) vary from slight tingling in the forearm and hand to complete paralysis in the area supplied by the compressed nerves. In the early manifestations the symptoms are increased and diminished by changing the position of the arm. Elevation of the shoulder and arm relieves the nerve pressure, and the patients find that they rest more comfortably at night with the arm above the head, and that the boring neuralgic pains and numbness are relieved by elevating the shoulder and supporting the body on the elbows. As the pressure becomes more severe and continued, the paræsthesia and paresis are followed by anæsthesia and paralysis. The numbness and tingling announce the existence of pressure for months or even years before paralysis develops. The muscles of the forearm fatigue on very slight exertion. These cases are most frequently diagnosed as brachial neuralgia, neuritis, etc. Lilienthal[6] quotes a case in which, in addition to pains of a cramp-like character radiating down the arm to the finger-tips the patient

[1] *Ann. Surg.*, 1898, xxviii. 509. [2] *Ibid.*, 1905, xli. 399.
[3] *Ibid.*, 1907, xlv. 638. [4] *Ibid.*, 1906, xliv. 630.
[5] *Ibid.*, 1905, xlii. 123. [6] *Ibid.*, 1905, xli. 766.

also complained of palpitation of the heart, occasional difficulty in swallowing, and of a girdle-like sensation about the throat. The change in the circulation is peculiar and striking, and involves the arterial, never the venous, current. If the patient presses his hands firmly together there will be a rapid return of circulation to the " life glow " on the normal side, while on the diseased side the hand remains blanched, wrinkled and cold from a minute to a minute and a half, resembling the prodromal pallor of impending Raynaud's disease. Cold water may thus feel many degrees colder on the affected than on the healthy side. There is no evidence of venous stasis. This is explained by the fact that the subclavian vein passes in front of the scalenus anticus, whereas the artery passes over the rib and behind the muscle, and may be kinked or flattened over the rib or compressed between it and the muscle. Where the pressure had been great and continuous, records of cases show that an obliterating endarteritis took place, and in some cases was followed by gangrene. More often, however, although no radial pulse could be felt, the anastomotic circulation was sufficient for the nutrition of the limb. Murphy suggests that when it was not so there had probably been a sudden and severe trauma of the artery in its exposed position which might produce thrombosis and necrosis. The sudden trauma of the artery could weaken its walls so that an aneurism would be produced, and this has undoubtedly occurred. But the diagnosis of aneurism should not be accepted in these cases except where a dissection and exposure of the artery has been made.[2] Owing to the artery being carried more forward, and its pulsations being more prominent, this error in diagnosis has frequently been made. In Murphy's case he says he could scarcely convince himself that aneurism was not present on account of the broad pulsation, as well as lateral expansion signs. The broad surface was, however, due to the flattened condition of the subclavian artery over the wide terminus of the rib. The bruit was present and extended in a downward direction, more marked with the shoulder depressed. With depression there is frequently an absent or diminished radial pulsation ; when the shoulder is elevated the pulsation again becomes normal. In a number of cases radial and brachial pulsation were absent, and did not return after the relief of pressure by removal of the rib. In Lilienthal's case the left pulse was completely obliterated on deep inspiration. There was a bruit over the left subclavian artery which ceased on deep respiration, and reappeared with increased loudness after holding the breath. In addition to the errors in diagnosis already referred to, the rib itself has been mistaken for an exostosis, osteo-sarcoma, or other tumour. Eisendrath[1] gives an epitome of thirty-four cases,

[1] *Amer. Medicine*, 1904, viii. 322.

on twenty-one of which operations were performed. In two of the cases, aged 15 and 17 respectively, there were no symptoms. The results of the operations were on the whole satisfactory, though it is obvious that it is a difficult proceeding. In several cases the pleura was wounded, and in two or three the nerve symptoms were temporarily increased by the operation. In almost all where the radial pulse was obliterated this was unaltered by the operation, and in some of the cases no improvement took place in paralysis affecting portions of the hands. As a rule an incision was made over the swelling in the neck. Lilienthal split the fibres of the sterno-mastoid, and Murphy says he made his incision at the posterior border of the scalenus anticus. The subclavian artery was displaced forwards, and the nerves of the brachial plexus inwards. Great care has to be taken in separating the nerves from the rib, as they are very liable to injury, as indicated by increase in the nervous symptoms immediately after operation. It is very important to remove the periosteum with the rib. The writer knows of a case where this was not done, and the rib was reproduced with return of symptoms. The writer has also had another case recently under his observation the details of which are as follows :—

Male, æt. 40, nine months ago complained of pain and numbness down right arm ; found the affected arm colder than the other, and thought it became very fatigued after using it for a short time. There was wasting of the arm, and of the muscles of the thenar eminence, with weakness of the arm, and complete absence of the radial pulse. A lump and pulsating tumour were formed above the clavicle. He had been treated for aneurism with iodide of potassium, etc., with relief to his symptoms, and he did not wish for an operation. Murphy says " all cases should be operated as soon as diagnosed."

**Wounds of the thoracic duct** during operation are sufficiently rare to be of interest. The writer has recently had a case in his own practice. After the removal of a malignant growth from the root of the neck, which was firmly adherent to the lower end of the internal jugular vein on the left side, a flow of clear thin fluid was observed from the depths of the wound. Numerous attempts were made to find the leaking spot and clamp it with forceps. For a long time these were unsuccessful. A spot was finally caught which appeared to arrest the flow, and this was ligatured, and the wound closed. During the succeeding twenty-four hours the dressings were saturated with discharge, and at the end of the second day a fluctuating swelling about the size of a hen's egg had appeared under the lower end of the sterno-mastoid. The patient complained of hoarseness of voice and a pain in the right side of the chest. A dressing was applied very firmly with an elastic bandage, and there followed some swelling of the arm and some blueness of the hand and left side of the face. Within a week the swelling was gone, there having been no discharge from the wound for a day or two before this, and the patient soon recovered completely with very little upset. The healing of the wound was delayed somewhat. De Forest[1] in a paper on this subject points out that these injuries are not nearly so fatal as used formerly to be supposed. He mentions two deaths in 31 cases, and certainly one of these may have been due to other causes than the injury to the duct. It appears that if the flow of chyle can be stopped (and it generally can be some way or other) the patient is most likely to recover, and furthermore this is likely to happen even if there has been leakage for a considerable time, and some emaciation has followed. In experiments upon animals it was found in dogs that when the duct was completely occluded there was merely a temporary impairment of nutrition, but that if the duct were cut through without ligaturing it a profuse chylorrhœa occurred, which if not checked will lead to progressive loss of strength and finally to death. The anatomy of the duct is not thoroughly understood, and probably is subject to great variation. There is no doubt that Nature has provided anastomosing channels by which the chyle can be carried into the general circulation after injury of the main duct. Sometimes the latter opens into the vein by many openings, as in the delta of a river; and when an injury occurs it is improbable that all these channels are damaged, but only the main trunk, so that the others are capable of dilatation and carrying on the chyle. Most of the cases have shown a flow of milky fluid at the time of operation, but in some this has not occurred until two or three days later. In many circumscribed œdema or a fluctuating swelling has occurred in the neck, and in some (as in my own case) œdema of the left side of the face and left arm. In many cases following tamponade of the duct the patient complains of a pressure in the thorax. De Forest submits that the correct treatment of a wounded thoracic duct is the same as that of a wounded artery. He says the following methods of treatment must be considered: (1) ligature, (2) suture, (3) suture of the enclosing tissues, (4) application of an artery clamp, (5) compression by firm tamponade. Preference should be given to that method which will certainly check the chylorrhœa at once. Finally the following conclusions are drawn:—

(1) The thoracic duct probably has collateral branches always which are able, in case of accident, to perform the function of the main duct. Further anatomical investigation concerning this are greatly to be desired.

(2) The sudden closure of the duct in man has had as its result only transitory disturbance in the nutrition of the body.

[1] *Ann. Surg.*, 1907, xlvi. 705.

(3) Chylorrhœa occurring after a wound of the duct must, if possible, be immediately controlled.

(4) The wounded thoracic duct may be treated precisely as we would treat a wounded blood vessel.

(5) Suture is the ideal method. If it is technically possible, its use is to be preferred, since the duct then remains patent.

(6) In all cases where suture is impossible a ligature should be applied. If ligature is technically impossible then in order of value, suture of the tissue, application of clamps, and in emergency as a last resort tamponading should be applied.

HAROLD F. MOLE.

